# New technique for the management of anteriorly located midline prostatic cysts causing severe lower urinary tract symptoms: Case report and literature review

**DOI:** 10.1016/j.ijscr.2019.01.019

**Published:** 2019-01-29

**Authors:** Khalid Abdulrahman AL-Nasser, Raed Mohammed Almannie

**Affiliations:** Division of Urology, Department of Surgery of Medicine and King Saud University Medical City, King Saud University, Saudi Arabia

**Keywords:** Anterior prostatic cyst, Prostate, Cyst, Obstruction, Lower urinary tract symptoms, Case report

## Abstract

**Introduction:**

Prostatic cysts are uncommon and usually asymptomatic. In most cases, these cysts are found incidentally by imaging. In some cases, these cysts can be symptomatic and missed for years.

**Case report:**

We report the case of a 36-year-old male patient presented with a 3-year history of weak urine stream, dysuria, frequency and urgency. Ultrasound, computed tomography and magnetic resonance imaging demonstrated a prostatic cyst measuring 1.5*1.2 cm occupying the bladder outlet and causing obstruction. Transverse incision of the cyst provided a satisfactory resolution of the patient’s complaint.

**Conclusion:**

Incision of a symptomatic midline anterior prostatic cyst is a safe and effective management option with no potential risk of retrograde ejaculation.

## 1 Introduction

Prostatic cysts are uncommon and are usually found incidentally. They are asymptomatic in 95% cases. The majority of prostatic cysts originate in the posterior area of the prostate as embryological remnants. They can be categorized based on their location, shape, embryogenic origin, interconnection with the prostatic urethra or seminal vesicles and presence of sperm in the cyst. They have traditionally been classified as Mullerian duct cysts, utriculus prostatic cysts, ejaculatory duct cysts, seminal vesicle cysts and prostatic retention cysts [1,2].

Midline prostatic cysts (MPCs) are extremely rare, with an incidence of less than 1% [3]. However, due to the recent advances in imaging modalities, such as computed tomography (CT), transrectal ultrasound (TRUS) and magnetic resonance imaging (MRI), their diagnosis has become more prevalent (5%–8.6%) [4,5]. Although the majority of patients with MPCs are asymptomatic and diagnosed incidentally, some may suffer obstructive or irritative voiding symptoms, recurrent urinary tract infections, epididymitis, chronic pelvic pain syndrome, hematospermia, low semen volume or even infertility [6,7]. Symptoms occur due to compression of adjacent structures, including the bladder neck, urethra and seminal vesicles. Additionally, small cysts can be misdiagnosed as benign prostatic hyperplasia (BPH) or neuropathic bladder [8].

We report the case of a 36-year-old male patient with no other medical conditions who presented with a 3-year history of severe lower urinary tract symptoms due to an anteriorly located prostatic cyst. The work has been reported in line with the SCARE criteria [9].

## 2 Case report

A 36-year-old male patient with no other medical conditions presented to our outpatient clinic with a history of weak urine stream, dysuria, frequency and urgency for the preceding 3 years. The patient was diagnosed in another hospital with prostatitis and given a full course of ciprofloxacin resulting in no improvement. His medical history was not significant in terms of previous urinary tract infections, urethral catheterization, perineal trauma or ejaculatory issues.

His International Prostate Symptom Score (IPSS) was 22, while his score for quality of life due to urinary symptoms was 5. A digital rectal examination revealed a firm, nontender prostate without palpable nodules. Urine analysis results were normal, and culture was sterile. Urine cytology was not suggestive of malignancy. His serum prostatic-specific antigen (PSA) level was 0.875 mcg/l. Other biochemical laboratory examinations were within normal ranges. The maximum flow rate was 6 ml/s with a flat curve.

**Fig. 1:**
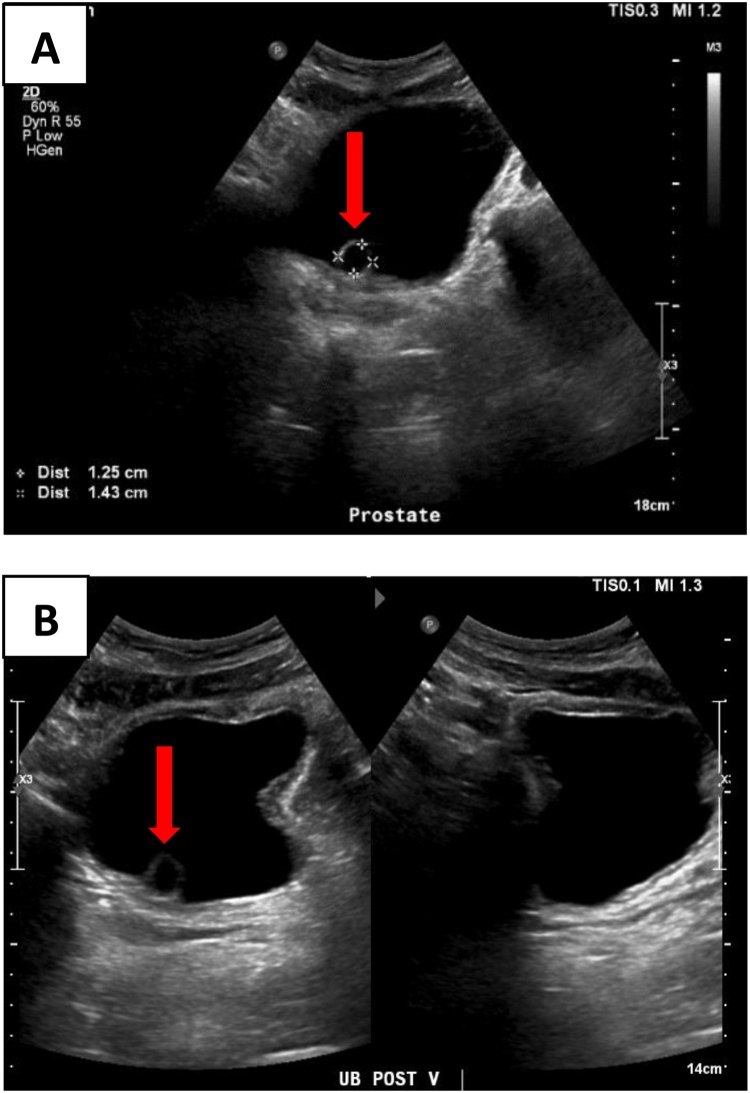
Pelvic ultrasound revealed a cyst measuring approximately 1.5*1.2 cm that was likely associated with the proximal part of the prostate gland and in close proximity to the proximal urethra (red arrow).

Pelvic ultrasound revealed a cyst measuring 1.5*1.2 cm that was most likely associated with the proximal part of the prostate gland. The full volume of the urinary bladder was 476 ml, and the postvoiding residual volume was 127 ml. The prostate gland was 38 g (Fig. 1).

**Fig. 2:**
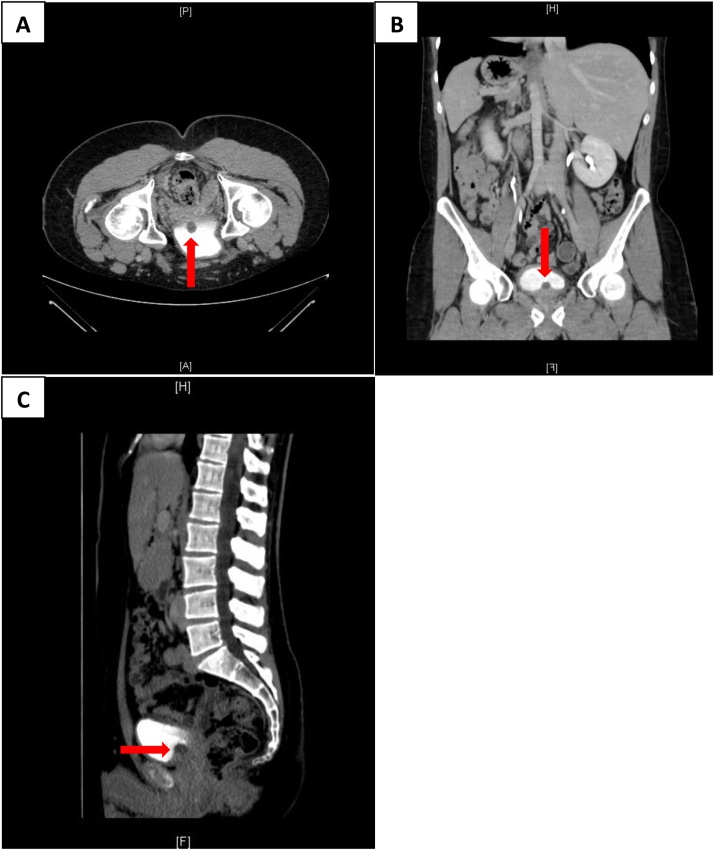
Axial **(A)**, coronal **(B)** and sagittal **(C)** CT urography images showing a low-density cystic lesion measuring 1.5*1.4 cm in size at the midline of the upper part of the bladder neck region indicating an anteriorly positioned prostatic cyst (red arrow).

CT urography was performed to exclude an ectopic ureterocele. A prostatic cyst measuring 1.5*1.4 cm in size was present at the midline of the upper part of the bladder neck region (Fig. 2).

**Fig. 3:**
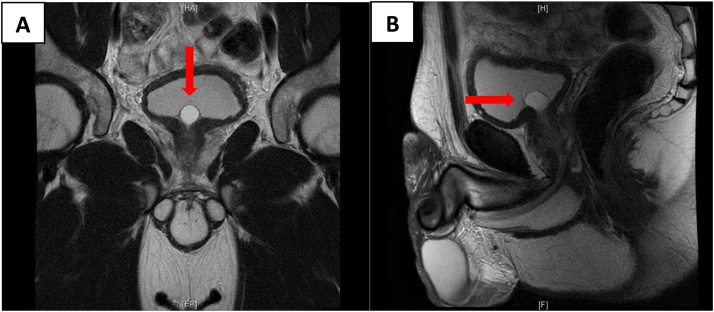
Coronal **(A)** and sagittal **(B)** pelvic MR images of the male patient showing a well-defined midline cyst arising from the prostatic base and herniating into the urinary bladder with no clear communication with the urethra. The cyst measured 1.6*1.3 cm and exhibited bright T2 signal intensity and hypo intense T1 signal intensity with peripheral wall enhancement characteristic of a cyst (red arrow).

MRI revealed a prostatic cyst measuring 1.6*1.3 cm with no clear communication with the urethra (Fig. 3).

**Fig. 4:**
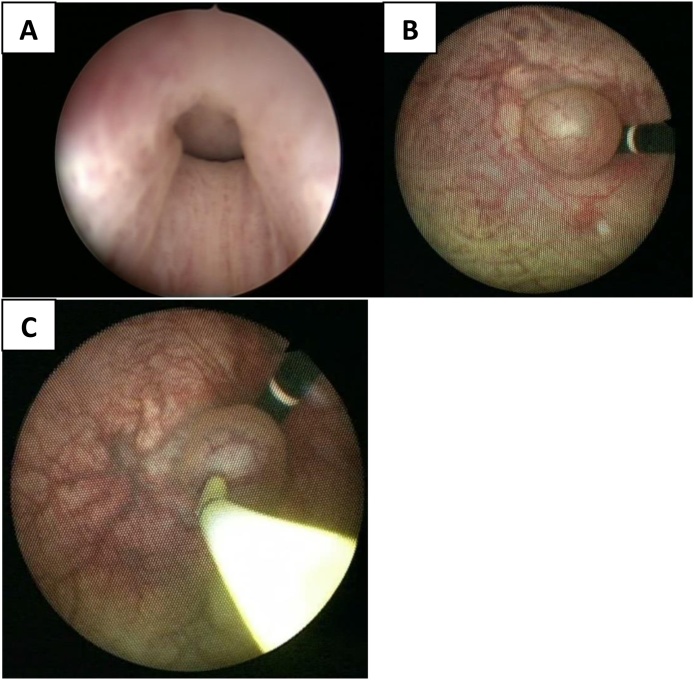
**(A)** and **(B)** an anteriorly positioned midline prostatic cyst obstructing the bladder outlet via an action similar to a ball-valve.

The patient was scheduled to undergo transurethral resection of the cyst. Under general anesthesia, the patient underwent cystourethroscopy with a 17 French flexible cystoscope. The cyst was obstructing and located at the bladder neck. Due to the age of the patient and the potential risk of retrograde ejaculation, a decision was made to incise the cyst. Retroflexion of the cystoscope clearly revealed the cyst (Fig. 3-B). Therefore, using retroflexion of the cystoscope, a transverse incision was performed in the superior part of the cyst using Holmium Laser. No intraoperative complications were observed (Fig. 4-C).

Postoperatively, the patient complained of minor hematuria and dysuria, which resolved a few days later. The patient’s lower urinary tract symptoms resolved immediately. His last follow-up examination at six months postoperatively showed no recurrence of lower urinary tract symptoms.

## 3 Discussion

Cystic lesions in the lower male genitourinary tract are uncommon and are typically benign. In some cases, they may be associated with various genitourinary abnormalities and symptoms, such as chronic pelvic pain syndrome, postvoiding incontinence, urinary tract infection, obstructive and/or irritative voiding symptoms, recurrent epididymitis, prostatitis, hematospermia, ejaculatory pain, low semen volume or even infertility in some cases [6,[10], [11], [12]].

The majority of prostatic cystic lesions are asymptomatic. They can cause symptoms depending on their anatomical relationship and size or when accompanied by infection. Most symptomatic prostatic cysts are located laterally [13]. Tambo et al. [14] examined 34 patients with symptomatic prostatic cysts and found that 40% of patients had obstructive urinary symptoms, 33% suffered from urinary retention, approximately 9% exhibited dysuria, and 6% were infertile.

Galosi et al. [15] reported that 9.8% of MPC cases can be detected by using TRUS. MPCs have also been reported to occur in 7.6% of healthy men and 5% of symptomatic outpatients [11]. However, imaging modalities such as CT, TRUS and MRI have increased the diagnosis rate of MPCs (5%–8.6%) [4,5].

In 2009, Galosi AB classified prostatic cystic lesions into 6 different types based on TRUS and their pathological correlations to: isolated medial cysts, cysts of the ejaculatory duct, simple or multiple parenchymal cysts, complicated cysts (infectious or hemorrhagic), cystic tumors and secondary cysts related to parasitic disease. MPCs are less common and located posteriorly in the majority of cases. They have traditionally been classified as Müllerian duct cysts, utriculus prostatic cysts, ejaculatory duct cysts, seminal vesicle cysts and prostatic retention cysts [2,13,15]. MPCs are further classified according to the presence of sperm in the fluid content using concomitant TRUS-guided opacification and dye injection. If sperm are present in the fluid content, then the cyst is assumed to be communicating with the seminal tract. Furuya et al. [12] further classified MPCs into four categories: Type 1 MPC, which has no communication with urethra (traditional prostatic utricle cyst); Type 2a MPC, which also has no communication with the urethra [cystic dilatation of prostatic utricle (CDU)]; Type 2b CDU, which has communication with the seminal tract; and Type 3 cystic dilatation of the ejaculatory duct. However, this classification is not used in clinical practice because all cysts have similar presentations and treatments [16].

Multiple therapeutic options have been described for symptomatic prostatic cysts, such as transrectal aspiration with or without sclerotherapy, marsupialization with a transurethral technique and open surgery in some cases [14,17]. Recurrence of cysts has been reported in some cases of incompletely excised cysts during open surgery. However, transurethral techniques have shown no recurrence of MPCs during follow-up. Zhang et al. [18] also recommended transurethral unroofing of small prostatic cysts that are close to the urethra or bladder cavity because of its simplicity, outcomes, low complication rate and short recovery period.

We searched for similar case reports of anteriorly located prostatic cysts causing lower urinary tract symptoms in different databases (MEDLINE, LILACS and Embase) using the key words “prostatic cyst”, “midline”, “lower urinary tract symptoms” and “case reports” and found a few published cases. The search was conducted on October 4, 2018 (Table 1).

**Table 1:** Literature search in different medical databases for case reports on “anteriorly located midline prostatic cyst arising from the bladder neck causing severe lower urinary tract symptoms in young male patient”.

Database	Search strategies	Papers found
MEDLINE	(prostatic cyst [Title]) AND lower urinary tract symptoms [Title]	3
	((midline [Title]) AND prostatic cyst [Title]) AND case reports [Publication Type]	3
	((midline [Title]) AND prostatic cyst [Title])	8
Embase	midline prostatic cyst [Case report]	1
LILACS	prostatic [Words] and cyst [Words] and case reports [Publication type]	5
	midline [Words] and prostatic [Words]	3

Although approximately 40 cases of symptomatic prostatic cysts have been reported, only 9 case reports of anteriorly located MPCs causing severe lower urinary tract symptoms have been published. All cases were done using either resectoscope or Laser with rigid scope.

Our patient was mainly suffering from obstructive voiding symptoms. A standard transurethral resection of the prostatic cyst was not performed due to the difficult position of the cyst and the potential risk of retrograde ejaculation. We describe a new technique to manage difficult located MPC. Transverse incision with laser of MPC using retroflexion of the cystoscope provided a satisfactory resolution of the patient’s complaint.

## 4 Conclusion

Incision of a symptomatic midline anterior prostatic cyst is a safe and effective management option with no potential risk of retrograde ejaculation.

## Conflicts of interest

Declared none.

## Sources of funding

Declared none.

## Ethical approval

Study is exempt from ethnical approval.

## Consent

Consent was made, patient is aware that his condition is very rare and managed by new technique, patient is satisfied after treatment.

## Author’s contribution

Khalid Abdulrahman AL-Nasser

- Writing manuscript.

- Literature review.

Raed Mohammed Almannie

- Supervising each step.

## Registration of research studies

Study has no registration number.

## Guarantor

Khalid Abdulrahman AL-Nasser.

## Provenance and peer review

Not commissioned, externally peer-reviewed.
